# Satisfaction des bénéficiaires du régime obligatoire d'assurance maladie des agents publics et assimilés à Lomé, Togo

**DOI:** 10.11604/pamj.2019.33.29.17291

**Published:** 2019-05-15

**Authors:** Tchaa Abalo Bakai, Didier Koumavi Ekouevi, Essotoma Beweli, Jean Iwaz, Anne Thomas, Nagham Khanafer, Kariyiare Goilibe, Esseboe Sewu, Yao Kassankogno, Nicolas Voirin

**Affiliations:** 1 Centre Africain de Recherche en Épidémiologie et en Santé Publique (CARESP), Lomé, Togo; 2 Hospices Civils de Lyon, Pôle Santé Publique, Service de Biostatistique- Bioinformatique, Lyon, France; 3 Université de Lyon, Lyon, France; 4 Université Lyon 1, Villeurbanne, France; 5 CNRS UMR 5558, Laboratoire de Biométrie et Biologie Évolutive, Équipe Biostatistique-Santé, Villeurbanne, France; 6 Epidemiology and Modelling, Dompierre-sur-Veyle, France; 7 Université de Lomé, Faculté des Sciences de la Santé (FSS), Département de Santé Publique, Lomé, Togo; 8 Institut National d'Assurance Maladie (INAM), Lomé, Togo; 9 Hospices Civils de Lyon, Hôpital Édouard Herriot, Service d'Hygiène, Épidémiologie et Prévention, Lyon, France

**Keywords:** Assurance maladie, satisfaction de l’usager, Togo, Health insurance, user satisfaction, Togo

## Abstract

**Introduction:**

En février 2011, le gouvernement togolais a instauré un régime obligatoire d'assurance maladie au profit des agents publics et assimilés. Quatre ans plus tard, cette étude a enquêté sur la satisfaction des bénéficiaires de ce régime dans la commune de Lomé.

**Méthodes:**

L'enquête a été réalisée auprès d'un échantillon de bénéficiaires à l'aide d'un questionnaire anonyme administré en face-à-face à la sortie de huit établissements de soins. Une analyse des réponses a permis de mesurer le degré de satisfaction. Les données quantitatives ont été décrites à l'aide de médianes et d'étendues interquartiles (EIQ) et les données qualitatives transcrites verbatim.

**Résultats:**

Parmi 288 sujet sollicités, 279 ont accepté de participer dont 58% étaient des femmes et 88% des agents encore en activité. L'âge médian était de 38 ans (EIQ: 30-47). Une très grande majorité de répondants (93,5%) a considéré cette assurance maladie « satisfaisante ». La note moyenne de satisfaction globale était de 6/10 (min: 5, max: 9). Les principaux éléments d'insatisfaction concernaient le refus de prise en charge des affections de longue durée (84% d'insatisfaits), la complexité des formalités administratives (84% d'insatisfaits), et les longueurs des délais de prise en charge (67% d'insatisfaits).

**Conclusion:**

Le niveau de satisfaction très élevé encourage le maintien et le développement de ce régime d'assurance maladie au Togo. Toutefois, les éléments d'insatisfaction devraient faire l'objet d'une prompte attention et de véritables efforts de correction.

## Abstract (en)

**Introduction:**

In February 2011, the Togolese Government established a compulsory health insurance for public officials and assimilated employees. Four years later, this study investigated the satisfaction of beneficiaries of this insurance plan in Lomé.

**Methods:**

The survey was conducted among a sample of beneficiaries using an anonymous face to face questionnaire outside eight health care facilities. The analysis of survey responses allowed measuring the degree of satisfaction. Quantitative data were described using median and extended interquartile range (IQR) and qualitative data were transcribed verbatim.

**Results:**

Out of 288 subjects invited to participate, 279 agreed to participate of whom 58% were women and 88% were officials in active employment. The average age of the participants was 38 years (IQR: 30-47). A very large majority of respondents (93.5%) considered this health insurance “satisfactory”. The mean overall satisfaction score was 6/10 (min: 5, max: 9). The main elements of dissatisfaction involved the refusal of care to those people with long-term illnesses (84% dissatisfied), the complexity of administrative formalities (84% dissatisfied) and care delays (67% dissatisfied).

**Conclusion:**

The very high level of satisfaction promotes the maintenance and the development of this health insurance in Togo. However, the elements of dissatisfaction should receive prompt attention and genuine efforts should be made to correct them

## Introduction

L'assurance maladie est une branche de la sécurité sociale chargée d'assurer à un individu la totalité ou une partie des dépenses de soins en cas de maladie et un revenu minimum en cas d'arrêt de travail. Dans les pays développés, les questions d'assurance maladie font depuis longtemps partie du débat public: ceci n'est pas le cas dans tous les pays aux revenus faibles et intermédiaires. Selon l'Organisation Mondiale de la Santé (OMS), près de la moitié de la population mondiale n'a toujours pas accès à des soins de santé de base et cette exclusion touche principalement les populations aux revenus très limités [1]. En Afrique, nombre de pays ont des régimes d'assurance maladie peu développés et des dépenses publiques de santé très limitées. Dans ces pays, le payement direct des soins constitue une importante partie des revenus des ménages [2-4]. Une amélioration de cette situation requiert une réflexion sur le financement de l'accès aux soins par la mise en place de régimes d'assurance maladie viables. Au Togo, suite à un processus participatif initié en 2009, la loi N°2011-003 du 18 février 2011 a institué un régime obligatoire d'assurance maladie au profit des agents publics et assimilés [5]. Le lancement officiel de ce projet a eu lieu le 5 septembre 2011 et l'Institut National d'Assurance Maladie (INAM) a été désigné pour en assurer le fonctionnement avec le principal objectif de permettre aux bénéficiaires une meilleure accessibilité à des soins de qualité [6]. Pour le gouvernement togolais, le développement de l'assurance maladie représente d'abord une possibilité durable pour les bénéficiaires de se faire soigner à temps et, ensuite, un moyen de lutter contre la pauvreté et d'atteindre les Objectifs du Millénaire pour le Développement (OMD) [6,7]. Quatre ans après l'instauration de ce régime obligatoire d'assurance maladie, il était important de l'évaluer auprès des usagers. L'objectif de la présente étude était de chercher le degré de satisfaction des bénéficiaires du régime dans la commune de Lomé, de repérer les motifs de satisfaction et d'insatisfaction et de rapporter les attentes des bénéficiaires.

## Méthodes

**Population et échantillonnage**: cette étude transversale descriptive a été réalisée auprès d'un échantillon de bénéficiaires du régime obligatoire d'assurance maladie. L'enquête s'est déroulée du 26 mai au 31 août 2015 à Lomé. La population étudiée était composée de bénéficiaires du régime (agents publics et assimilés en activité ou à la retraite) des deux sexes, âgés de 18 ans et plus, ayant consulté, au moins une fois, un agent prescripteur et disposant d'une pièce justificative (attestation de soins, attestation de remboursement de soins, attestation d'actes médicaux ou d'analyses biologiques). Ces bénéficiaires ont été recrutés dans huit établissements de soins de la commune de Lomé ayant signé un partenariat avec l'INAM. Il s'agissait des deux Centres Hospitaliers Universitaires (CHU Sylvanus Olympio et CHU Campus), des deux Centres Hospitaliers Régionaux (CHR Lomé commune, Hôpital de Bè) et de quatre centres médico-sociaux (CMS Agoè-Nyévé, CMS Bè-Atikoumé, CMS Cacavéli et CMS Adidogomé). L'étude a donc concerné tous les établissements de niveau I et II et quatre établissements de niveau III tirés au sort parmi les 17 établissements de Lomé.

**Taille de l'échantillon**: le nombre de répondants nécessaires à cette étude a été calculé sur la base des critères suivants: 1) pourcentage de satisfaits de l'ordre de 75% (p) pour une précision (i) de 5%, avec un risque de première espèce alpha (α) de 5%. La taille de l'échantillon a été calculée à l'aide de la formule suivante:

$$n=\text{p}\left(1-\text{p}\right)\frac{1,96}{{\text{i}}^{2}}$$

Ainsi, 288 répondants étaient nécessaires pour cette étude. L'enquête a donc concerné 36 patients de chaque établissement et a sollicité, systématiquement, chaque troisième patient sorti des consultations (raison de sondage régulière).

**Recueil des données**: l'étude a utilisé un questionnaire anonyme de 20 items administré oralement par un enquêteur à chaque participant. Outre ces items fixes, le questionnaire comportait un encart vide destiné à recueillir l'expression libre de chaque participant. Les enquêteurs ont été recrutés localement parmi des étudiants en médecine inscrits à la Faculté des Sciences de la Santé de l'Université de Lomé et formés aux techniques et à la pratique des entretiens (qualité d'écoute, influence réciproque entre questions et réponses, création d'un climat de confiance entre enquêté et enquêteur). Les données recueillies concernaient les caractéristiques socio-démo-économiques des participants, les services offerts et les remboursements, le système d'information et d'orientation, et quelques caractéristiques des relations régime-bénéficiaires (confiance, fiabilité, rapidité, équité).

**Analyse des données**: Les données du questionnaire ont été saisies et analysées avec le logiciel Epi-info version 7. Les données quantitatives ont fait l'objet d'une analyse descriptive. Elles ont été décrites à l'aide de médianes et d'étendues interquartiles (EIQ). Les données qualitatives (verbatim) ont fait l'objet d'une analyse de contenu thématique. Le niveau de satisfaction des participants a été considéré comme l'indicateur principal de leur perception de la qualité des services.

**Éthique et réglementation**: cette étude a obtenu l'accord des autorités administratives et sanitaires de la Ville de Lomé, de l'INAM et de la Faculté des Sciences de la Santé. L'objet et le déroulement du questionnaire ont été expliqués à chaque candidat à la participation. Un formulaire de consentement libre et éclairé à la participation a été signé par chaque participant. Cette signature était précédée, si nécessaire, par une interprétation orale des éléments du formulaire en dialecte Mina. Chaque enquêteur a signé un accord de confidentialité.

## Résultats

L'étude a sollicité 288 patients bénéficiaires du régime d'assurance. Elle a exclu neuf patients qui ont interrompu l'entrevue peu après son démarrage. L'analyse a donc retenu 279 participants (taux de réponse: 97%).

**Caractéristiques des répondants**: la majorité des répondants (58,1%) étaient de sexe féminin et leur âge médian était de 38 ans (EIQ: 30-47). Parmi ces répondants, 91% avaient suivi des études secondaires ou universitaires, 88% étaient encore en activité et 80% étaient mariés (Tableau 1).

**Tableau 1:** Distribution des 279 participants selon les principales caractéristiques

Caractéristique	Effectif (%)
**Âge**	
<38 ans	150 (53,8)
38-58 ans	100 (35,8)
59-78 ans	29 (10,4)
**Sexe**	
Femme	162 (58,1)
Homme	117 (41,9)
**Statut matrimonial**	
Célibataire	39 (13,9)
Marié	222 (79,6)
Divorcé	15 (5,4)
Veuf	3 (1,1)
**Niveau d'étude**	
Jamais scolarisé	1 (0,4)
Primaire	25 (8,9)
Secondaire	117 (41,9)
Supérieur	136 (48,8)
**Situation professionnelle**	
En activité	245 (87,7)

**Les points de satisfaction**: à Lomé, une très grande majorité des répondants (93,5%) était satisfaite du régime obligatoire d'assurance maladie du Togo. Pour 84,6% d'entre eux, le régime assurait efficacement la couverture des frais liés à la maladie et aux accidents et, pour 83,5%, les services offerts par le régime répondaient effectivement à leurs attentes. La note moyenne de satisfaction globale était de 6/10, la note minimale de 5 et la note maximale de 9.

**Satisfaction des participants**: le Tableau 2 présente la satisfaction des participants du fonctionnement du régime d'assurance maladie en matière d'offre de soins. Le service « tiers payant » a été jugé très satisfaisant (25,2% des répondant) ou satisfaisant (64,4%). Le taux de cotisation (prélèvement de 3,5% du salaire) a recueilli 66,3% d'opinions favorables. Le taux de remboursement (80% et 100% en fonction des maladies) et la prise en charge des ayants droit ont été jugés satisfaisants par 81,9% et 76,4% des répondants. Se sont révélés également satisfaisants les remboursements des actes suivants: suivi de la grossesse et de l'accouchement (71,6%), analyses biologiques et imagerie médicale (64,9%), hospitalisation (62,7%), vaccins et dispositifs médicaux (61,2%), et actes chirurgicaux et paramédicaux (58,1%).

**Tableau 2:** Dégré de satisfaction des 279 participants du fonctionnement du régime obligatoire d’assurance maladie, Lomé, Togo

	Satisfaction		Insatisfaction	
Fonctionnement ou prestation	Service très satisfaisant	Service satisfaisant	Service peu satisfaisant	Service pas du tout satisfaisant
Les relations du régime avec les bénéficiaires	1,8	43,4	44,8	10,0
Les réunions d'information organisées	0,7	14,7	48,8	35,5
Les formulaires et les imprimés	0,7	15,1	53	31,2
Le processus de prise en charge en cas de maladie	1,1	32,3	46,2	20,4
Les délais de prise en charge	2,5	30,5	50,5	16,5
Le service tiers payant	25,2	64,4	9,0	1,4
Le taux de cotisation fixé à 3,5% du salaire	8,6	57,7	27,6	6,1
Le taux de remboursement de 80% à 100%	16,8	64,9	16,1	2,2
Les prestations sous condition d’entente préalable	2,1	16,9	41,2	39,8
Le processus d’obtention d’une entente préalable	0,7	6,1	50,9	42,3
La prise en charge des affections de longue durée	6,1	30,5	50,5	12,9
Le processus d'obtention de l'attestation de traitement d’une pathologie chronique	1,8	13,9	59,9	24,4
La liste des prestations exclues	1,4	20,1	44,8	33,7
La prise en charge des ayants droit (enfants, conjoint)	18,3	58,1	17,5	6,1
Le nombre maximum de personnes prises en charge fixé à 6	13,3	55,6	22,9	8,2
L'âge limite de prise en charge fixé à 21 ans pour les enfants	5,4	36,2	32,9	25,5
Le service de prise en charge soins/hospitalisation	3,6	59,1	30,8	6,5
Le service de prise en charge des actes médicaux ou chirurgicaux	2,5	55,6	33,3	8,6
Le service de prise en charge des analyses biologiques	3,2	61,7	29	6,1
Le service de prise en charge de la grossesse et de l’accouchement	8,2	63,4	24,1	4,3
Le service de prise en charge des vaccins et des dispositifs médicaux	5,7	55,5	29,1	9,7
L’équité	2,2	67,7	24,4	5,7
L’accessibilité, la continuité, et le suivi de la prise en charge	0,7	63,4	30,2	5,7

**Figure 1:**
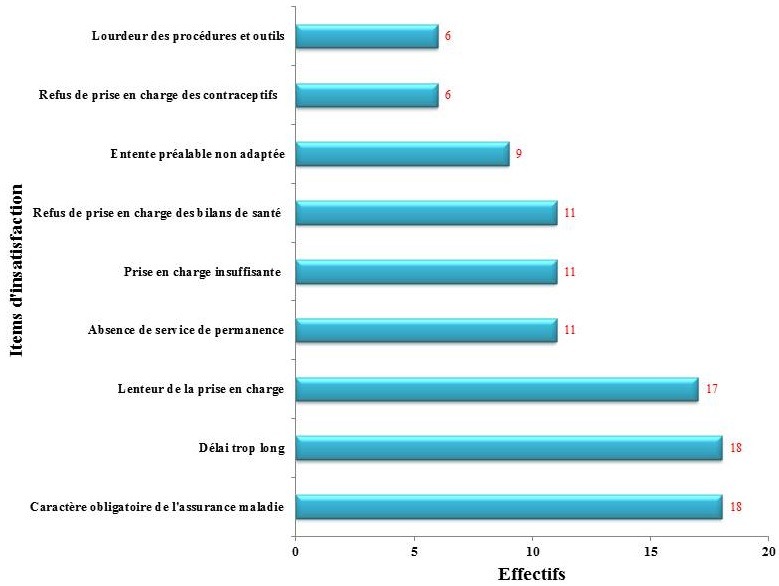
Points d´insatisfaction de 107 participants à l´enquête de satisfaction du régime obligatoire d´assurance maladie des agents publics et assimilés à Lomé, Togo

**Insatisfaction des participants**: par contre, certaines prestations ou certains fonctionnements étaient considérés non satisfaisants (peu satisfaisants ou pas du tout satisfaisants) (Tableau 2): les procédures d'obtention de l'entente préalable (93,2%), les procédures d'obtention d'attestation pour le traitement des pathologies chroniques (84,3%), les réunions d'information organisées (84,3%), les formulaires et les imprimés (84,2%), la liste des soins soumis à entente préalable ou exclus (81% et 78,5%), les délais de prise en charge (67%), les décisions de prise en charge des affections de longue durée (63,4%) et les relations régime-bénéficiaires (54,8%). Parmi les 288 participants, 107 ont accepté de s'exprimer librement à la fin du questionnaire guidé. Ces participants ont exprimé des insatisfactions concernant le caractère obligatoire de l'assurance, les longs délais de remboursement, les lenteurs à la prise en charge ainsi que d'autres aspects importants du fonctionnement du régime (Figure 1).

## Discussion

En Afrique, les études qui évaluent la satisfaction des bénéficiaires d'un régime d'assurance maladie sont très peu nombreuses et très peu présentes dans la littérature scientifique francophone. La présente enquête de satisfaction est la première conduite au Togo depuis le lancement du régime obligatoire d'assurance maladie dans ce pays (fin 2011). Elle n'a été commanditée ni par l'INAM ni par l'État togolais mais conduite à l'initiative du Département de Santé Publique (Faculté des Sciences de la Santé, Université de Lomé) dans le cadre des recherches sur l'amélioration de la qualité des soins offerts à la population togolaise. Ses résultats sont attendus pour éclairer les gestionnaires du régime sur les réformes qu'il convient de lui apporter. Cette enquête a bénéficié d'un très fort taux de participation (97%) qui s'explique probablement par le mode personnel du recueil des données, par le mode de recrutement des participants ainsi que par l'intérêt des participants pour le régime d'assurance. Ce taux ainsi que le recrutement du nombre nécessaire donne à cette étude une bonne crédibilité. La légère prédominance des femmes s'explique par la plus vive attention des femmes aux problèmes de santé personnelle et familiale au Togo, comme ailleurs.

La participation à la partie « expression libre » du questionnaire a été néanmoins faible (37%); elle peut s'expliquer par la réserve des répondants. Toutefois, elle peut aussi s'expliquer par l'exhaustivité du questionnaire; en effet, plusieurs remarques émises en expression libre se trouvaient déjà parmi les rubriques du questionnaire et avaient été mentionnées comme « insatisfaisantes » par les participants. Malgré la satisfaction globale, la note moyenne de satisfaction ne fut pas très élevée (6/10). Précisions ici que la note moyenne de satisfaction et le taux de satisfaction rapportés dans cette étude sont proches de ceux obtenus en France en 2009 (6/10 et 93,5% vs. 7/10 et 92%) [8]. Cette étude a montré un fort taux de satisfaits (93,5%). Toutefois, ce taux doit être interprété avec circonspection parce qu'un avis favorable dans une enquête ne dit pas nécessairement que le service reçu est satisfaisant mais simplement que rien de très grave ne s'est produit [9]. Cette circonspection et la note moyenne de 6 devraient porter l'attention des gestionnaires du régime aux principaux motifs d'insatisfaction tels que la complexité de certaines démarches et la liste des soins soumis à entente préalable. Au moment de la conception du protocole de cette enquête, les participants devaient être interrogés à leur domicile ou sur leur lieu de travail parce qu'il a été prouvé que l'expression d'un patient est plus libre et plus critique chez lui qu'à la sortie d'un hôpital [10]. Mais, finalement, les enquêtes ont été menées face-à-face à la sortie des établissements de soins en raison de difficultés logistiques et financières.

Malgré un recrutement consécutif des participants, le mode d'échantillonnage a pu constituer un biais de sélection en raison de sa limitation dans le temps. De plus, il est difficile d'exclure un biais de mémoire relatif à des expériences antérieures. Néanmoins, le «biais de désirabilité sociale» a été minimisé en expliquant à chaque répondant qu'il n'y a ni bonne ni mauvaise réponse. Le mode d'administration du questionnaire en face-à-face a permis un taux de participation très élevé (97%) et donc une meilleure confiance dans les résultats. Cette administration du questionnaire a également minimisé le «biais de non-réponse» (lorsque l'analyse ne tient pas compte des personnes qui refusent de participer à l'enquête) qui pouvait «conduire à surestimer le niveau de satisfaction, argument souvent évoqué lors de telles enquêtes» [11]. Les résultats de cette enquête réalisée à Lomé ne peuvent être généralisés à l'ensemble des bénéficiaires du Togo. Cependant, la concentration sur la capitale reflète les résultats d'une région où l'accès aux soins est le meilleur et l'application du régime la meilleure. La satisfaction dans les autres régions serait probablement inférieure d'où l'importance de mener des enquêtes décentralisées pour vérifier cette hypothèse.

## Conclusion

Cette première investigation au Togo a confirmé qu'une grande majorité (93,5%) des participants était satisfaite du fonctionnement du régime obligatoire d'assurance maladie des agents publics et assimilés. Malgré ses limites, cette enquête a fourni des données fiables et suffisantes pour plaider pour l'extension progressive de ce régime à d'autres catégories sociales, voire à toute la population: c'est la Couverture Sanitaire Universelle, principe de base des soins de santé primaires rénovés de l'OMS de 2008. Les nombreuses remarques recueillies sont précieuses pour situer les points d'insatisfaction et pour hiérarchiser les attentes des bénéficiaires et y répondre.

### État des connaissances actuelles sur le sujet

- À ce jour, peu d'enquêtes de satisfaction ont été menées en Afrique sur des régimes d'assurance maladie facultatifs ou obligatoires;
- Avec certaines précautions, les enquêtes de satisfaction peuvent indiquer les points d'insatisfaction qui nécessitent analyse et amélioration.

### Contribution de notre étude à la connaissance

- 93,5% des participants assurés au régime obligatoire d'assurance maladie des agents publics et assimilés de Lomé (Togo) se sont déclarés satisfaits de ce régime;
- Ces participants ont soulevé, en même temps, plusieurs domaines d'insatisfaction qu'il convient d'améliorer;
- Cette satisfaction encourage les gestionnaires de ce régime à l'étendre à d'autres secteurs de la population en vue d'atteindre la Couverture Sanitaire Universelle. Avec ses avantages et ses inconvénients, cette enquête peut servir de support à d'autres enquêtes africaines dont les résultats enrichiraient les connaissances sur les attentes des africains en matière de couverture sanitaire.

## Conflits d’intérêts

Les auteurs ne déclarent aucun conflit d’intérêts.
